# Dynamic formation of stable current-driven plasma jets

**DOI:** 10.1038/s41598-019-39827-6

**Published:** 2019-02-22

**Authors:** Thomas C. Underwood, Keith T. K. Loebner, Victor A. Miller, Mark A. Cappelli

**Affiliations:** 1 High Temperature Gasdynamics Laboratory, Stanford University, Stanford, California 94305 USA; 2 SeekOps, Inc., Austin, Texas 78745 USA

## Abstract

Instabilities play a prominent role in determining the inherent structure and properties of magnetized plasma jets spanning both laboratory and astrophysical settings. The manner in which prominent unstable modes dynamically evolve remains key to understanding plasma behavior and control. In astrophysical phenomena, self-similar jets are observed to propagate over vast distances while avoiding breakup caused by unstable mode growth. However, the production of stable dense plasma jets in the laboratory has been limited by the onset of unstable modes that restrict jet lifetime, collimation, and scalability. In this work, we visualize the formation of stable laboratory-generated, dense, super-magnetosonic plasma jets in real time, and we identify an underlying mechanism that contributes to this behavior. The current-driven plasma jets generated in our experiments form a flowing Z-pinch, which is generally unstable to the m = 1 kink instability. Our results indicate that a stable dense plasma jet can be maintained for timescales over which a steady pinch current can be sustained, even at levels which would otherwise lead to rapid unstable mode growth and resultant pinch disassembly.

## Introduction

The dynamics of unstable mode growth is a principal concern in understanding plasma behavior and control. In particular, current-driven plasma jets exhibit a host of instabilities and yet retain remarkable features of practical interest. For instance, unstable modes play a role in the formation and propagation of astrophysical jets^1–4^ and are a limiting factor in plasma-jet driven magnetized target fusion^5,6^, z-pinch schemes^7–9^, and spheromak and compact toroid plasma generation and control^10–12^. Understanding how stable plasma jets can be formed and maintained remains an important area of ongoing research. Recent work in both the pinch^13–15^ and tokamak^16^ communities indicate that background shear flow can significantly contribute to plasma stability. In this work, we present direct schlieren visualizations of current-driven plasma jets and use this diagnostic to view their stable formation in real-time. We observe that magnetohydrodynamic (MHD) instabilities initially present in the system are dynamically damped over observable scales as the plasma flow fully develops. Finally, we present a theoretical description to identify the unstable modes and quantify the role that shear flow plays in the observed MHD stability of plasma jets.

**Figure 1:**
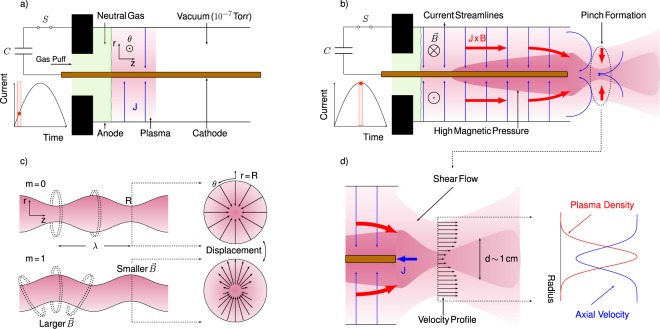
Operational Theory: Schematic detailing the underlying physics responsible for producing jets in a current-driven pulsed plasma accelerator. (**a**) Details how the neutral gas is ionized and accelerated. (**b**) Shows how an axial z-pinch is formed at the end of the accelerator. (**c**) Illustrates the MHD instability modes that classically dominate similar systems. (**d**) Shows the resulting density and velocity profiles within the pinch structure of the flow.

Current-driven plasma jets are generated using a pulsed plasma accelerator (Fig. 1). The apparatus features a coaxial electrode volume that is 26 cm long and 5 cm in diameter, and accelerates a plasma armature to Alfvénic velocities^17,18^ under the influence of the self-induced Lorentz force. A 56 *μ*F capacitor bank, which can be charged to between 5 and 9 kV, supplies energy to the flow.

To initiate a plasma jet, a puff valve injects hydrogen gas into vacuum (10^−7^ Torr) at the entrance of the accelerator. The working gas breaks down and conducts a radial current between the cathode and anode to form a plasma armature wherein the current is driven by the bank voltage. The return current along the central electrode generates an azimuthal magnetic field, which accelerates the plasma armature in a deflagration wave as neutral gas continues to be supplied, ionized, and accelerated during the 10–20 *μ*s pulse duration^19–21^.

At the exit plane of the electrode volume, the self-generated azimuthal magnetic field exerts a radial Lorentz force on the canted current streamlines of the emerging plasma jet. Figure 1c) depicts the plasma compression caused by the radial Lorentz force, which forms a hot, dense pinch at the core of the jet. The accelerated plasma surrounding the pinch maintains a quasi-steady axial shear flow featuring characteristic velocities of 10^5^ m/s, while the axial flow velocity through the pinch itself is comparatively lower due to its position in the wake of the central electrode^22^. The resulting dense plasma jet is inherently unstable to both the *m* = 0 ‘sausage’ and *m* = 1 ‘kink’ MHD modes; however, a stable jet is observed for timescales over which a steady current is sustained.

We observe the structure of the plasma jet using schlieren imaging, a refractographic technique that is capable of visualizing a transparent medium. The signal constrast, Δ*I*/*I*, of the diagnostic can be expressed in terms of the index of refraction, *N*, of the medium according to,

1 $$\frac{{\rm{\Delta }}I}{I}\propto \int \frac{1}{{N}}\frac{{\rm{\partial }}{N}}{{\rm{\partial }}y}dx,$$

where *y* corresponds to the direction of the optical cutoff and *x* is the path over which the laser propagates^23,24^. Thus schlieren imaging visualizes the path integrated first derivative of a medium’s refractive index as a gradient in recorded intensity. In a neutral gas, the refractive index is directly related to the gas density, which can vary to values slightly above unity as a result of compressible flow phenomena such as shocks, expansion waves, and thermally-driven density gradients^23^. In a typical plasma system, the neutral gas density increases the refractive index above unity, whereas the free electron density provides a large negative contribution to the refractive index. In this work, the plasma densities in the jet core and pinch approach 10^23^ m^−3^ ^21^; thus, we produce a nearly fully ionized plasma wherein the contributions to the refractive index from neutral atoms are negligible, and the refractive index gradients resulting from the electron density variations alone are sufficiently large so as to be readily visible in schlieren images.

**Figure 2:**
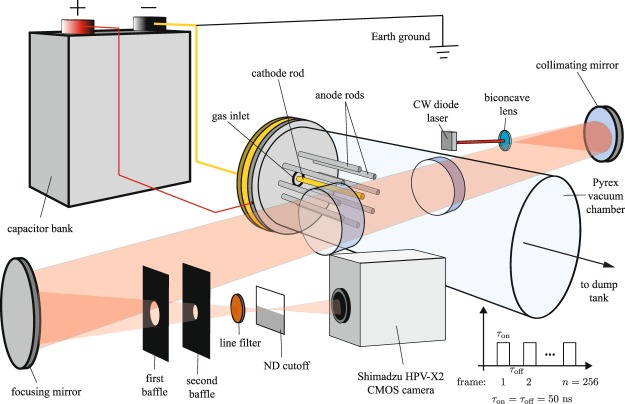
Experimental Setup: Experimental setup used to visualize the flow using the z-type schlieren imaging technique at 10 MHz. A laser backlight is used to overcome plasma self-emission and a sooted slide is used as an optical cutoff in place of a conventional knife-edge to limit diffraction effects.

We capture the dynamic evolution of the jet structure using an ultra-fast camera that records 256 consecutive frames at a 10 MHz repetition rate with 50 ns exposures. During each exposure, the distance traversed by the plasma sets the axial spatial resolution of the diagnostic, Δ*x* ∼ *V*Δ*t*_exp_ ∼ 0.5 cm, for *V* = 10^5^ m/s based on experimental time-of-flight measurements^25^ and Δ*t*_exp_ = 50 ns. A purpose-built, transparent vacuum chamber was constructed for the purpose of creating these visualizations, as depicted in Fig. 2. The Methods section further details the implementation of the cinematic schlieren diagnostic including features used to maximize sensitivity while minimizing diffraction effects.

## Results

### Experimental Visualization

**Figure 3:**
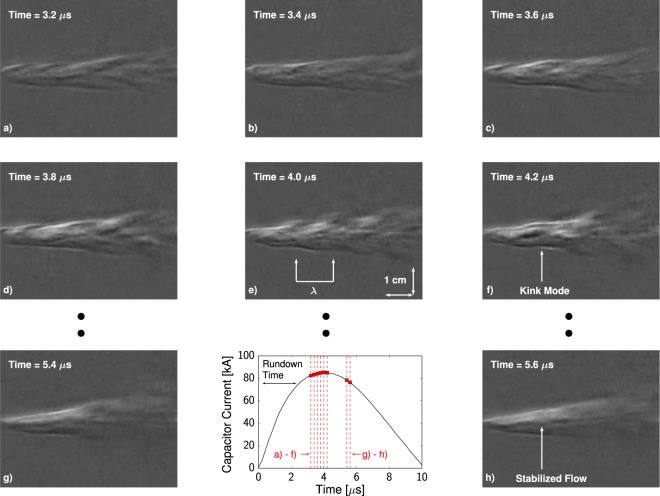
Schlieren Image Sequence: Schlieren images of the plasma jet with an external energy supply of 2.3 kJ at select times with 50 ns exposures. The flow is observed to be initially unstable to long-wavelength MHD instabilities that appear as perturbations on the envelope of the plasma column. At later times and with higher current flowing through the pinch, the flow appears to be stabilized over the observed window.

Figure 3 depicts a set of schlieren images with a vertical optical cutoff corresponding to select times during a pulse driven by 2.3 kJ of stored energy. The Supplementary Information includes the remaining frames spanning the entire discharge duration. Following the time delay required to accelerate the plasma down the gun volume (∼2 *μ*s), a broad axial pinch attaches to the central cathode at the exit of the accelerator. At early times after pinch formation, a number of coherent, long-wavelength instabilities appear, resembling the ‘kink’ MHD modes depicted in Fig. 1. These instability modes are observed to shift the entire plasma column vertically in a quasi-periodic manner. To capture their spatial characteristics, the quasi-periodic ‘features’ that result in radial perturbations of the plasma column are identified and tracked over time, an example of which is shown in Fig. 3. The tracked modes are prevalent early in the jet evolution, specifically 3.5–4.5 *μ*s with between 3–8 features tracked per frame, and evolve to have peak axial wavelengths ranging from *λ*_*z*_ ≈ 1.2–2.2 cm. As these dominant length scales are more than twice the axial spatial resolution, measurements indicate that the shorter wavelengths are being damped within the flow. At later times during the discharge, the surface perturbations of the plasma column disappear, indicating a stable plasma jet has formed.

**Figure 4:**
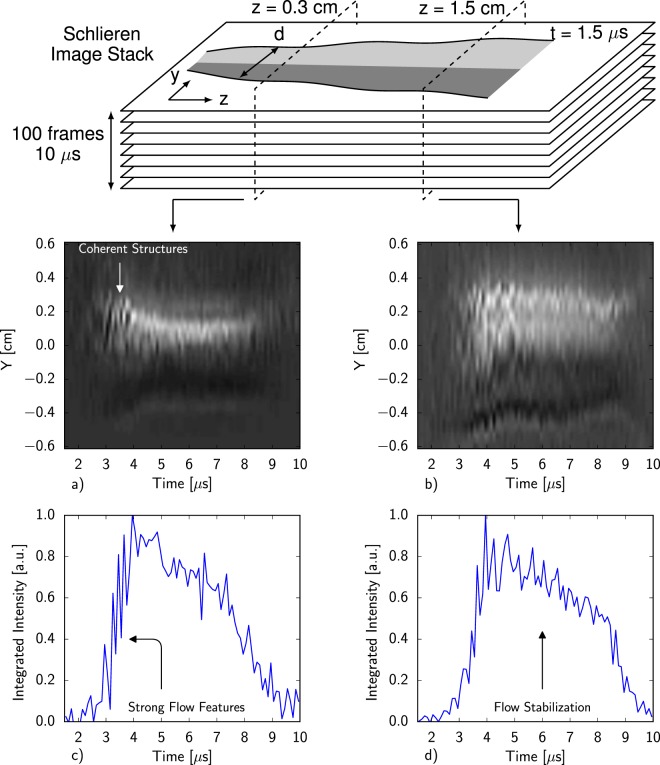
Jet Formation Process: Streak images of the plasma jet at fixed axial locations (**a**,**b**) formed by taking vertical slices of the schlieren image stack. Integrated schlieren signals of the top half of each of the streak images (**c**,**d**) illustrate that the inhomogeneities in the plasma column that are initially prevalent within the jet decrease in frequency over time.

To illustrate the formation process of the plasma jet, vertical slices of the jet at fixed axial locations are organized into streak images depicted in Fig. 4. At both 0.3 cm and 1.5 cm axially downstream of the electrodes, the prevalence and amplitude of both streaks and inhomogeneities in the plasma column decrease over the lifetime of the jet. Integration over the top half of the streak images, shown in Fig. 4, also indicate that the high amplitude oscillations in the schlieren signal during the initial formation of the jet (2–5 *μ*s) are damped in time. A stable jet is maintained until a sudden expansion around 8.5 *μ*s when the drive capacitors no longer supply any current to sustain the quasi-steady flow. Although the drive current as measured at the capacitor bank is smaller once a stable jet forms, *in situ* measurements detailed in ref.^26^ indicate that the current flow within the pinch peaks 2–4 *μ*s later and is thus greater than at earlier times when instabilities are more prevalent. The phase lag between the current passing through the pinch and the drive current is also demonstrated by the fact that the effective radius of the pinch decreases over the lifetime of the jet. Thus, the radial Lorentz force, and therefore axial current increases over time, while the observed perturbations decrease over time. Since the pinch appears to become more stable as the current supplied to it is increased, these results indicate the possibility of increasing both the duration and current density of the discharge to produce relatively long-lived stable plasma jets.

### Stability Analysis

We carry out a linear analysis of the ideal MHD equations to identify the instabilities observed experimentally in Fig. 3 from 3.5–4.5 *μ*s and quantify the role that flow properties have on the resulting stability spectrum. The ideal limit of these equations is taken as the fastest timescales of the system are of specific interest in our analysis. To justify this assumption, we note that for conditions of interest in the core of the plasma, the ratio of the resistive and Alfvénic timescales approaches ∼100. The quantitative basis for this assertion including specific plasma properties is included in the Methods section. Without loss of generality, the Eulerian primitive form of the perturbed variables (*ρ*_1_, ***v***_1_, *p*_1_, and ***B***_1_) are recast in terms of the plasma displacement vector ***ξ*** to reduce system dimensionality^27^. After normal mode analysis has been performed in time, ***ξ***(***r***,*t*) = ***ξ***(***r***)*exp*(−*iωt*), the system of equations can be written as,

$$\omega {\rho }_{0}{\boldsymbol{\xi }}={\rho }_{0}{\boldsymbol{u}}-i{\rho }_{0}{{\boldsymbol{v}}}_{0}\cdot \nabla {\boldsymbol{\xi }}$$

$$\omega {\rho }_{0}{\boldsymbol{u}}=-\,i\omega {\rho }_{0}{{\boldsymbol{v}}}_{0}\cdot \nabla {\boldsymbol{\xi }}-G({\boldsymbol{\xi }})$$

where $G({\boldsymbol{\xi }})={{\boldsymbol{J}}}_{1}\times {{\boldsymbol{B}}}_{0}+{{\boldsymbol{J}}}_{0}\times {{\boldsymbol{B}}}_{1}-\nabla {p}_{1}-{\rho }_{0}{({{\boldsymbol{v}}}_{0}\cdot \nabla )}^{2}{\boldsymbol{\xi }}+\nabla \cdot [{\boldsymbol{\xi }}({\rho }_{0}{{\boldsymbol{v}}}_{0}\cdot \nabla {{\boldsymbol{v}}}_{0})]$ is the generalized force operator and subscripts indicate the equilibrium (0) and perturbed quantities (1). A variable substitution has been introduced to reduce the order of the resultant stability equation and to recast the system in terms of the characteristic eigenvalue equation *ωA*⋅***x*** = *B*⋅***x***. This facilitates the use of linear eigenvalue solvers to calculate the resultant spectrum^28,29^. We solve the equations in a cylindrical geometry, and assume mode decomposition in *θ* and *z* according to ${\boldsymbol{\xi }}({\boldsymbol{r}})=({\xi }_{r}(r)\,\hat{{\boldsymbol{r}}}+{\xi }_{\theta }(r)\hat{{\boldsymbol{\theta }}}+{\xi }_{z}(r)\hat{{\boldsymbol{z}}})\exp (im\theta +ikz)$. The radial variation of each vector component is discretized using mixed finite element basis functions according to the Appert projection^30^ to minimize spectral pollution. This analysis approximates the jet as an axisymmetric plasma column with uniform axial properties. Further details about the numerical implementation are included in the Supplementary Information.

**Figure 5:**
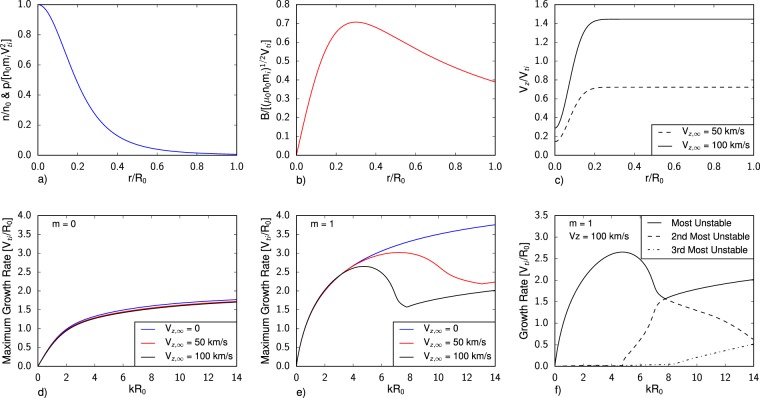
Stability Analysis: Results of the linear stability analysis of current-driven plasma jets. (**a**–**c**) Details of the equilibrium Bennett profiles used to model the axial pinch formed in the jet. (**d**,**e**) Show the *m* = 0 and *m* = 1 instability mode calculations as a function of *k*, the axial wavenumber, while (**f**) shows how the dominant spectral modes vary over *k*. Within the plots, *n*_0_, *R*_0_, and *V*_z,∞_ are the are the peak number density, radius, and free stream velocity respectively in the simulated profile and ${V}_{ti}=\sqrt{2{k}_{b}T/{m}_{i}}$ is the ion thermal velocity where a temperature of *k*_*b*_*T* = 25 eV was assumed^22^.

The equilibrium profiles used in the analysis, shown in Fig. 5a–c and detailed further in the Methods section, are determined from an assumed analytic Bennett profile given plasma density and velocity fields specified from a combination of experiments and numerical simulation. At the edge of the domain, a vacuum boundary condition closes the system of equations. Nominal plasma density and temperature are obtained from experimental Stark broadening measurements^21^ and simulations^22^ respectively. The shape of the density profile is inferred from Abel inverted density profiles in ref.^25^. The axial velocity profile dips from a value of V_z,∞_ in the surrounding flow to a value of 0.2V_z,∞_ on axis just downstream of the central cathode, consistent with detailed resistive MHD simulations of the accelerator geometry^22^ that show higher velocity plasma is not entrained in the central pinch. The peak velocity of the simulations are also consistent with time-of-flight measurements made by feature tracking of the leading edge of the plasma plume, as highlighted in ref.^25^. The functional form and amplitude of the magnetic field is detailed in the Methods section and is inferred from equilibrium arguments of the flow configuration. Each calculation was performed using 1000 elements for a variety of peak axial velocities, azimuthal mode numbers, and axial wavenumbers. For each condition, the largest growth rates were recorded by tabulating the largest imaginary eigenvalues, ${\rm{\Gamma }}\equiv \Im (\omega )$, in the spectrum.

The results of the linearized stability analysis are detailed in Fig. 5d–f. Radial eigenmodes of the *m* = 1 azimuthal (kink) mode dominate the calculated spectrum and are sensitive to the presence of axial shear. For velocities consistent with those expected in experimental conditions, the most unstable kink mode reduces in amplitude and shifts to longer wavelengths, illustrating that axial shear plays an important role in determining the stability spectrum. At V_z,∞_ ∼ 100 km/s, *n* ∼ 10^23^ m^−3^, and *T* ∼ 25 eV, representative of conditions in Fig. 3, the most unstable mode peaks at *kR*_0_ = 4.7, where *k* = 2*π*/*λ*_*z*_. For a column radius of 1 cm, the corresponding dominant axial wavelength is 1.3 cm which is within range of the observed experimental disturbances. Given uncertainties in the exact flow properties during the time where instabilities are prevalent in Fig. 3, the agreement between experiment and the stability analysis adds further evidence that the observed perturbations on the plasma column include the classical ‘kink’ MHD modes. To quantify when viscous and resistive effects become important, we note that the critical scale when viscous dissipation timescales are equal to growth rates is *λ*_crit_ ∼ L^*^/Re^1/2^ ^31^. Taking the limiting value of Re  ∼ 10^25^ yields a critical scale of *kR*_0_ ∼ 19, outside the range of dominant modes of interest.

## Discussion

Our observations and analyses have shed light on the process by which stable current-driven plasma jets form. In our experiments, long-wavelength disturbances are dynamically damped over the observable window as the jet forms. At sufficiently high current densities in the central pinch, the plasma jet appears free of discernible long-wavelength instabilities. Analyses based on linearized ideal MHD equations are able to identify the initial characteristic instabilities as classical ‘kink’ modes and show their growth rate and spectrum are impacted by the presence of sheared axial flows. Importantly, experimental visualizations indicate that a stable, quasi-steady jet is produced in the laboratory through a dynamic process that involves the evolution of conventional MHD modes.

The findings summarized in this work have important implications for the study of plasma jets in nature, as well as the use of high energy density jets in terrestrial applications. This study motivates future time-resolved measurements of stability beyond a linearized analysis to uncover a detailed mechanistic understanding of flow transition and dynamic instability growth in hydromagnetic systems. Future efforts to measure the shear flow properties over time and develop fully nonlinear simulations will help to interpret the formation process of stable plasma jets that was visualized in this work. Furthermore, efforts to incorporate diagnostics capable of measuring azimuthal perturbations in the plasma column will allow further characterization of coherent structures in the flow and the process by which they are stabilized. With the refractographic diagnostic described in this study, it is possible to make novel dynamic measurements of plasma flow systems and in highly repeatable experimental facilities, expand this study to more comprehensively and quantitatively compare to theory.

This work is supported by the U.S. Department of Energy Grant No. DE-NA0002011. The authors would like to thank Todd Rumbaugh at Hadland Imaging for generously providing imaging equipment that made this work possible.

## Methods

### Description of Schlieren Experiment

A standard z-type schlieren configuration was employed with *f*/4, 60 cm focal length mirrors, as detailed in Fig. 2. Self-emission from the plasma is exceptionally bright, and overwhelmed several broadband schlieren light sources which were attempted (high-powered LED, spark lamp, etc.). To overcome this, we utilized a red continuous wave (CW) laser with a nominal output power of 250 mW paired with a narrow bandpass filter (centered at *λ* = 635 nm with FWHM = 2 nm). Schlieren imaging systems that use laser backlights or other coherent sources can suffer from diffraction effects, which manifest as fringes at the cutoff that appear as ring and streak artifacts in the generated images^32^. To mitigate these effects, a partial cutoff wherein the cutoff has a non-zero transmission fraction was used in lieu of a hard cutoff (i.e., a razor blade). This configuration provides a more gradual optical cutoff that dampens the diffraction modes in the resulting image. As a further noise-reduction measure, extensive optical baffling was installed between the camera and the test section to reject plasma self-emission and other stray light in order to elevate the schlieren signal above the background signal.

A high-speed Shimadzu HPV-X2 camera is used to record the schlieren signal with 50 ns exposures at a sample frequency of 10 MHz for the duration of the jet lifetime (up to 256 total frames). Image acquisition was triggered by the actuation of a gas valve occurring ∼500 *μ*s before breakdown, and delayed such that the image sequence begins roughly 10 frames before the plasma reaches the exit plane of the accelerator. The camera was fitted with an adjustable objective lens, and a spatial calibration object (i.e., a dot matrix) was imaged and analyzed in order to determine a projected pixel size of 0.237 mm/pixel or (0.119 mm/pixel of the video included in the Supplementary Information).

Despite all the efforts described above to eliminate diffraction effects and background signal, some artifacts were still present in the acquired images. To correct these aberrations and extract the maximum schlieren signal, a customized post-processing algorithm was developed and applied. A dark noise image was subtracted from each image, and the first 5 frames of the sequence (prior to any working gas entering the viewing area) were used to generate a background tare image that was subtracted from each of the subsequent frames. Lastly, we developed and implemented a temporal-and-spatial-frequency-domain filter for removing time-dependent ring and streak artifacts. Additional details on the post processing routine can be found in^32^.

### Description of Cinematic Schlieren Recording

A video of the schlieren sequence highlighted in Fig. 3 of the main text is included as a separate video file. A sample frequency of 10 MHz with 50 ns exposures was maintained over the duration of the jet’s lifetime spanning 12 *μ*s. The pulse length of the jet for this experiment configuration is 10 *μ*s.

### Finite Element Formulation

To form the characteristic matrix, we follow the traditional Galerkin approach of finite element by multiplying the MHD equations by some test functions, $\hat{{\boldsymbol{\xi }}}$ and $\hat{{\boldsymbol{u}}}$,

$${L}_{1}=2\pi {\int }_{0}^{R}\hat{{\boldsymbol{u}}}\cdot [\omega {\rho }_{0}{\boldsymbol{\xi }}-{\rho }_{0}{\boldsymbol{u}}+i{\rho }_{0}{{\boldsymbol{v}}}_{0}\cdot \nabla {\boldsymbol{\xi }}]rdr=\mathrm{0,}$$

$${L}_{2}=2\pi {\int }_{0}^{R}\hat{{\boldsymbol{\xi }}}\cdot [\omega {\rho }_{0}{\boldsymbol{u}}+i\omega {\rho }_{0}{{\boldsymbol{v}}}_{0}\cdot \nabla {\boldsymbol{\xi }}+G({\boldsymbol{\xi }})]rdr=0.$$

Integration by parts is performed on the MHD force operator that produces a boundary term to be evaluated. The resulting mathematical form can be written in a number of ways that are symmetric by construction. Following the mathematical development outlined in ref.^33^, this term can be written according to,

$$\begin{array}{ll}\int \hat{{\boldsymbol{\xi }}}\cdot G({\boldsymbol{\xi }})rdr & =-\int [\gamma {p}_{0}(\nabla \cdot {\boldsymbol{\xi }})\nabla \cdot \hat{{\boldsymbol{\xi }}}+{\boldsymbol{Q}}\cdot \hat{{\boldsymbol{Q}}}+\frac{1}{2}\nabla {p}_{0}\cdot ({\boldsymbol{\xi }}\nabla \cdot \hat{{\boldsymbol{\xi }}}+\hat{{\boldsymbol{\xi }}}\nabla \cdot {\boldsymbol{\xi }})+\ldots .\\  & \ldots \frac{1}{2}{{\boldsymbol{J}}}_{1}\cdot (\hat{{\boldsymbol{\xi }}}\times {\boldsymbol{Q}}+{\boldsymbol{\xi }}\times \hat{{\boldsymbol{Q}}})+\frac{1}{2}{\rho }_{0}({{\boldsymbol{v}}}_{0}\cdot \nabla {{\boldsymbol{v}}}_{0})\cdot ({\boldsymbol{\xi }}\cdot \nabla \hat{{\boldsymbol{\xi }}}+\hat{{\boldsymbol{\xi }}}\cdot \nabla {\boldsymbol{\xi }})-\ldots \\  & \ldots \rho ({{\boldsymbol{v}}}_{0}\cdot \nabla {\boldsymbol{\xi }})\cdot ({{\boldsymbol{v}}}_{0}\cdot \nabla \hat{{\boldsymbol{\xi }}})]rdr+{\rm{B}}.{\rm{T}}.\end{array}$$

where *γ* = 5/3 is the ratio of specific heats and B.T. signifies the boundary term. Additional variables referenced in the integration by parts form include,

$$\begin{array}{c}{\boldsymbol{Q}}=\nabla \times ({\boldsymbol{\xi }}\times {{\boldsymbol{B}}}_{0}),\\ \hat{{\boldsymbol{Q}}}=\nabla \times (\hat{{\boldsymbol{\xi }}}\times {{\boldsymbol{B}}}_{0}),\\ {{\boldsymbol{J}}}_{1}=\nabla \times \nabla \times ({\boldsymbol{\xi }}\times {{\boldsymbol{B}}}_{0})/{\mu }_{0}.\end{array}$$

The boundary condition in finite element is implemented by replacing the term arising from integration by parts with the vacuum perturbed pressure at the boundary *r* = *R*,

$${[{p}_{1}+{{\boldsymbol{B}}}_{0}\cdot {{\boldsymbol{B}}}_{1}/{\mu }_{0}]}_{r=R}={[{{\boldsymbol{B}}}_{0}\cdot {\tilde{{\boldsymbol{B}}}}_{1}/{\mu }_{0}]}_{r=R},$$

where ${\tilde{{\boldsymbol{B}}}}_{1}$ is the perturbed vacuum pressure. In the limit of no wall, Bessel functions can be employed to show that^34^,

$${{\boldsymbol{B}}}_{0}\cdot {\tilde{{\boldsymbol{B}}}}_{1}=-\,\frac{{k}_{\parallel }^{2}{\xi }_{r}{K}_{R}}{k{R}^{2}{\dot{K}}_{R}}{|}_{r=R}.$$

In the boundary condition, *k*_∥_ = *mB*_*θ*_ while *K*_*R*_ = *K*_*m*_(*kR*) represents the modified Bessel function. The projections outlined in ref.^30^ are used to change the variables and ensure the quantity ▽⋅***ξ*** has no truncation error,

$$\begin{array}{cc}{\xi }_{r}\, & =\{\begin{array}{cc}r{\xi }_{1} & {\rm{i}}{\rm{f}}\,m=0\\ {\xi }_{1} & {\rm{e}}{\rm{l}}{\rm{s}}{\rm{e}}\end{array}\\ {\xi }_{\theta }\, & =\{\begin{array}{cc}ir{\xi }_{1}-ir{\xi }_{2} & {\rm{i}}{\rm{f}}\,m=0\\ \frac{i{\xi }_{1}}{m}-\frac{ir{\xi }_{2}}{m} & {\rm{e}}{\rm{l}}{\rm{s}}{\rm{e}}\end{array}\\ {\xi }_{z} & \,=-\frac{i{\xi }_{3}}{k}\end{array}$$

The same form of the projection functions are also used for ***u***. The finite element expansions used for these new variables are,

2 $$\begin{array}{lll}{\xi }_{1}={e}^{im\theta +ikz}\sum {\xi }_{1j}{\varphi }_{1j}(r), & {\xi }_{2}={e}^{im\theta +ikz}\sum {\xi }_{2j}{\varphi }_{2j}(r), & {\xi }_{3}={e}^{im\theta +ikz}\sum {\xi }_{3j}{\varphi }_{3j}(r),\\ {u}_{1}={e}^{im\theta +ikz}\sum {u}_{1j}{\varphi }_{1j}(r), & {u}_{2}={e}^{im\theta +ikz}\sum {u}_{2j}{\varphi }_{2j}(r), & {u}_{3}={e}^{im\theta +ikz}\sum {u}_{3j}{\varphi }_{3j}(r),\end{array}$$

while the test functions take the form ${\hat{\xi }}_{\mathrm{1,}i}={e}^{-im\theta -ikz}{\varphi }_{1i}$ for each component of $\hat{{\boldsymbol{\xi }}}$ and $\hat{{\boldsymbol{u}}}$. In equation (2), *ξ*_*j*_ represent nodal values while *ϕ*_*j*_ represent the various basis functions within the domain. A combination of piecewise linear and piecewise constant functions are used to ensure that ∇⋅***ξ*** can be 0 to machine precision across any region,

$$\begin{array}{cc}{\varphi }_{1j} & \,=\{\begin{array}{cc}\frac{r-{r}_{j-1}}{{r}_{j}-{r}_{j-1}}, & {\rm{f}}{\rm{o}}{\rm{r}}\,{r}_{j-1} < r < {r}_{j}\\ \frac{{r}_{j+1}-r}{{r}_{j+1}-{r}_{j}}, & {\rm{f}}{\rm{o}}{\rm{r}}\,{r}_{j} < r < {r}_{j+1}\\ 0 & {\rm{o}}{\rm{t}}{\rm{h}}{\rm{e}}{\rm{r}}{\rm{w}}{\rm{i}}{\rm{s}}{\rm{e}}\end{array}\\ {\varphi }_{2j}\, & ={\varphi }_{3j}=\{\begin{array}{cc}1 & {\rm{f}}{\rm{o}}{\rm{r}}\,{r}_{j} < r < {r}_{j+1}\\ 0 & {\rm{o}}{\rm{t}}{\rm{h}}{\rm{e}}{\rm{r}}{\rm{w}}{\rm{i}}{\rm{s}}{\rm{e}}\end{array}\end{array}$$

### Equilibrium Profiles

The equilibrium profiles used as a part of the linear stability analysis follows from an analytic Bennett profile,

$$\begin{array}{c}{B}_{0}=\frac{r{B}_{m}}{a\mathrm{(1}+{(r/a)}^{2})},\\ {p}_{0}=\frac{\mathrm{(1}+\mathrm{1/}Z){n}_{0}(r=\mathrm{0)}{k}_{b}T}{{\mathrm{(1}+{(r/a)}^{2})}^{2}}\mathrm{.}\end{array}$$

*Z* defines the ionization state of the ions, ${B}_{m}=\sqrt{\mathrm{2(1}+\mathrm{1/}Z){\mu }_{0}{n}_{0}(r=\mathrm{0)}{k}_{b}T}$, and the parameter *a* controls where the radius of maximum magnetic field occurs. For conditions of our experiment, hydrogen gas was used as the working gas and thus *Z* = 1. The equilibrium density profile follows from pressure for a fixed plasma temperature *k*_*b*_*T*. Based on spatially resolved density profiles^25^ and comparisons to detailed MHD simulations^22^, this parameter was set to *a*/*R*_0_ = 0.3^22^. The analytic form of the velocity profile is given by,

$${V}_{z}={V}_{z,\infty }-{V}_{z\mathrm{,0}}\exp (-\frac{{r}^{2}}{{\gamma }^{2}}),$$

where *V*_*z*,∞_ represents the free stream velocity and both *V*_*z*,0_ and *γ* control the spatial structure. To be consistent with detailed numerical simulations of the geometry, parameters *V*_*z*,0_ = 0.8*V*_*z*,∞_ and *γ*/*R*_0_ = 0.1 where chosen for the quasi-steady velocity profile^22^ while *V*_*z*,∞_ was varied between 0 and 100 km/s.

### Timescales

Justification of the ideal MHD limit requires evaluation of both the resistive, *τ*_*r*_, and Alfvénic, *τ*_*A*_, timescales,

$${\tau }_{r}\sim \frac{{R}_{0}}{{V}_{A}},$$

$${\tau }_{A}\sim \frac{{\mu }_{0}{R}_{0}^{2}}{\eta },$$

where *R*_0_ is the characteristic pinch radius, *V*_*A*_ is the Alfvén velocity, *μ*_0_ is the permeability of free space, and *η* is the plasma magnetic resistivity. For a hydrogen plasma with *T* = 25 eV, the Saha equation predicts an ionization fraction approaching 1, so the resistivity for a fully ionized plasma can be expressed as,

$${\eta }_{\perp }[{\rm{\Omega }}\,{\rm{m}}]=1.039\times {10}^{-4}\frac{Z{\rm{\Lambda }}}{{T}_{e}^{\mathrm{3/2}}[eV]},$$

where Λ is the Coulomb logarithm and is further described in ref.^25^. Using this definition and noting for a hydrogen plasma, the Alfvén velocity follows, ${V}_{A}=B/\sqrt{{\mu }_{0}n{m}_{p}}$, the ratio of timescales can be evaluated. For conditions of interest to this paper, *n* ∼ 10^23^ m^−3^, *R*_0_ ∼ 1 cm, *T* ∼ 25 eV, and ${B}_{m}=\sqrt{\mathrm{2(1}+\mathrm{1/}Z){\mu }_{0}{n}_{0}(r=\mathrm{0)}{k}_{b}T}\sim 1.4$ T, and thus *τ*_*r*_/*τ*_*A*_ ≈ 150. Thus as *τ*_*r*_/*τ*_*A*_ ≫ 1, the dissipative timescales and quantities do not significantly contribute to the fastest and most unstable modes in the growth spectrum.

## Supplementary information

Schlieren Video
